# Retraction: Computational fluid dynamics modeling of the millisecond methane steam reforming in microchannel reactors for hydrogen production

**DOI:** 10.1039/d1ra90094c

**Published:** 2021-03-30

**Authors:** Junjie Chen, Xuhui Gao, Longfei Yan, Deguang Xu

**Affiliations:** Department of Energy and Power Engineering, School of Mechanical and Power Engineering, Henan Polytechnic University 2000 Century Avenue Jiaozuo Henan 454000 P. R. China comcjj@163.com +86-15138057627

## Abstract

Retraction of ‘Computational fluid dynamics modeling of the millisecond methane steam reforming in microchannel reactors for hydrogen production’ by Junjie Chen *et al.*, *RSC Adv.*, 2018, **8**, 25183–25200, DOI: 10.1039/C8RA04440F.

The authors have retracted this *RSC Advances* article in its entirety because there is considerable overlap with respect to the data presented in the previous article ‘Millisecond methane steam reforming for hydrogen production: a computational fluid dynamics study’ (*Int. J. Hydrogen Energy*, 2018, **43**, 12948–12969, DOI: 10.1016/j.ijhydene.2018.05.039) by the same authors. This means that the *RSC Advances* article is redundant. Despite low levels of textual overlap, much of the data is essentially the same as or very similar to that previously reported. The model was improved, but the degree of improvement is relatively low. There were some new findings presented, but the contribution is relatively small. Furthermore, the previous article was not cited in the *RSC Advances* article.

As much of the data are available in the previous article, the authors are retracting this *RSC Advances* article. All authors agree to this retraction.

Signed: Junjie Chen, Xuhui Gao, Longfei Yan, and Deguang Xu

Date: 18th March 2021

## Supplementary Material

